# Pediatric knee avulsion fractures: A systematic review of injury patterns, treatment strategies, and outcomes

**DOI:** 10.1007/s00068-025-02969-6

**Published:** 2025-09-23

**Authors:** Yasmin Youssef, Lukas F. Heilmann, Nils Mühlenfeld, Bastian Mester, Johannes Weber, Kai Fehske, Orla Klatte, Doreen Schneidmüller, Peter Strohm, Ralf Henkelmann

**Affiliations:** 1 Klinik für Orthopädie, Unfallchirurgie und Plastische Chirurgie, Universitätsklinikum Leipzig, Liebigstr. 20, Leipzig, 04103 Germany; 2 Klinik und Poliklinik für Unfallchirurgie und Orthopädie, Universitätsklinikum Hamburg-Eppendorf, Hamburg, Germany; 3 Klinik für Orthopädie und Unfallchirurgie, Universitätsklinikum Freiburg, Breisgau, Germany; 4 Klinik für Unfall-, Hand- und Wiederherstellungschirurgie, Universitätsklinikum Essen, Essen, Germany; 5 Klinik und Poliklinik für Unfallchirurgie, Universitätsklinikum Regensburg, Regensburg, Germany; 6 Klinik für Orthopädie und Unfallchirurgie, Johanniter-Waldkrankenhaus Bonn, Bonn, Germany; 7 Klinik für Unfall-, Hand-, Plastische und Rekonstruktive Chirurgie, Universitätsklinikum Würzburg, Würzburg, Germany; 8 Klinik für Orthopädie und Unfallchirurgie, Albertinen Krankenhaus Hamburg, Hamburg, Germany; 9 BG Unfallklinik Murnau, Murnau am Staffelsee, Germany; 10 Klinik für Orthopädie und Unfallchirurgie, Klinikum Bamberg, Bamberg, Germany

**Keywords:** Pediatric avulsion fractures, Avulsion fracture, Knee, Adolescent, ACL, Eminence fracture

## Abstract

**Background:**

The purpose of this systematic review was to analyze the existing literature on pediatric knee avulsion fractures regarding injury patterns, treatment strategies, and clinical outcomes. We aimed to identify evidence gaps and highlight the need for standardized diagnostic and therapeutic approaches for these clinically relevant injuries.

**Methods:**

The systematic review was registered on PROSPERO and conducted in accordance with PRISMA guidelines. A comprehensive search of PubMed and Cochrane databases was performed to identify peer-reviewed studies focusing exclusively on pediatric patients with avulsion fractures of the knee. Data on patient characteristics, treatment strategies, and clinical outcomes were extracted for qualitative analysis.

**Results:**

Eighty-three studies, comprising 1,676 patients (mean age 13.1 years), were included. The majority were case reports or case series, with an overall low level of evidence. Heterogeneity in treatment approaches, follow-up, and outcome measures limited comparability and precluded meta-analysis. Tibial eminence fractures (32.5%) and tibial tubercle fractures (18.1%) were most described, followed by rare injuries such as patellar avulsions, PCL and ACL avulsions. Surgical treatment is predominated, typically involving screw or suture fixation. Notably, tibial eminence fractures frequently presented with concomitant meniscal or chondral injuries (up to 40%), prompting recommendations for routine MRI diagnostics. Postoperative outcomes were generally favorable, with high rates of full range of motion and return to sport.

**Conclusion:**

Pediatric knee avulsion fractures show diverse injury patterns with limited evidence on optimal management. Despite generally favorable outcomes, data heterogeneity and the lack of standardized treatment underscore the need for multicenter prospective studies. Future research should aim to establish evidence-based diagnostic and therapeutic protocols.

## Introduction

Pediatric avulsion fractures of the knee are rare but diagnostically and therapeutically challenging injuries [1, 2]. Avulsion fractures occur when a bone fragment is torn away from the main bone by a tendon or ligament, as a result of acute tensile forces that surpass the strength of the bone’s attachment point [3, 4]. They usually occur during activities that involve sudden, forceful muscle contractions or joint motion [3]. The dynamic interplay between the developing musculoskeletal system and the forces exerted during physical activities renders children susceptible to specific patterns of avulsion injuries [2]. Especially adolescents seem to be susceptible to avulsion fractures due to their increase in muscle strength and the resulting relative weakness of the osteocartilaginous junctions, as well as due to hormonal changes. The knee joint is particularly susceptible to avulsion fractures due to its complex anatomy and the considerable forces it endures during physical activity [2]. Common sites for avulsion fractures in the pediatric knee include the tibial tubercle, the distal femur, the intercondylar eminence and the patella.

In children and adolescents, the presence of growth plates (physes) and the ongoing process of bone development contribute to the distinctive nature and implications in the successful therapy of these injuries compared to similar fractures in adults [1]. Prompt and accurate diagnosis of pediatric avulsion fractures is critical to prevent potential complications such as growth disturbances, chronic instability, and long-term functional impairment. Treatment approaches vary based on the location and severity of the fracture, ranging from conservative management with immobilization to surgical intervention for more severe cases [2]. While eminence fractures of the tibia and tibia tubercle fractures are comprehensively covered in the current literature and there are recommendations for their therapeutic management [5, 6], other rare occurring avulsion fractures of the knee, such as femoral tears of the cruciate ligament or tears of the collateral ligament, are underrepresented and there are only individual case reports and case series that describe their treatment [7–11]. There is no defined gold standard or algorithm for the diagnosis, treatment, and follow-up treatment for the care of pediatric patients with these avulsion fractures. Both conservative and operative options have been described [1, 2, 12, 13]. Further comprehension of these fractures is essential for optimizing patient care, improving outcomes, and fostering the development of targeted preventive strategies.

In the following review the current knowledge and recent advancements on the diagnosis and treatment options will be concisely summarized.

## Materials and methods

The presented study was registered on the International Prospective Register of Systematic Reviews (PROSPERO) network (CRD42023488550) before starting the review process and was performed accordingly to the Preferred Reporting Items for Systematic Reviews and Meta-Analyses (PRISMA) guidelines [14]. 

**Fig. 1:**
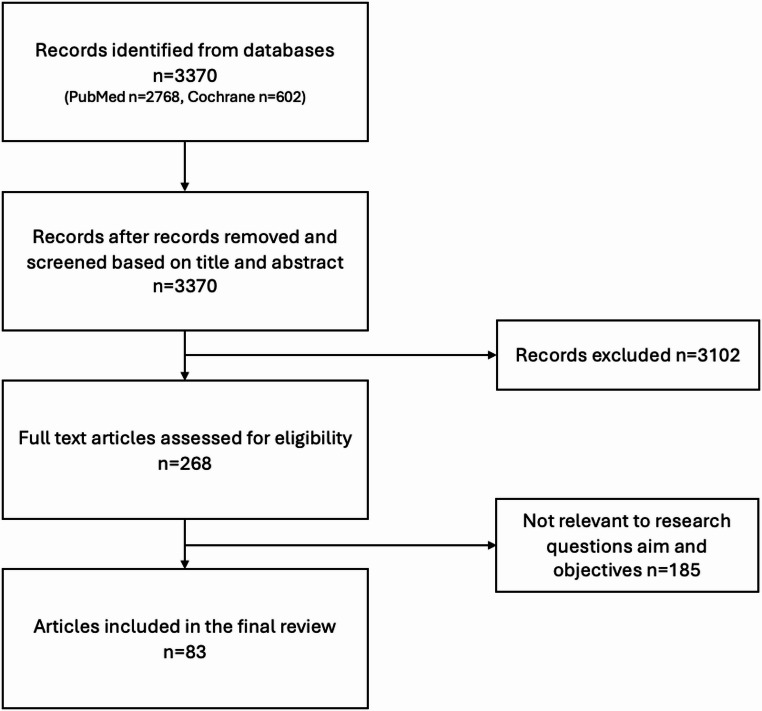
PRISMA flow diagram for the visual representation of the article selection process

A systematic literature search was conducted in the PubMed and Cochrane databases for all peer-reviewed publications published in English or German up to January 2024. Meta-Analyses, systematic reviews, narrative reviews, expert opinions and economic studies were excluded. However, reviews, editorials and opinion articles were screened as potential sources for additional references. Furthermore, studies which considered adult and pediatric patients in a combined cohort were excluded. The titles and abstracts of all identified studies were independently screened for eligibility by two independent authors (RH, YY) according to predefined inclusion and exclusion criteria. Subsequently, the full texts of potentially relevant articles were assessed for eligibility. The remaining studies were included for data extraction and qualitative analysis. In the event of disagreement during the selection process, the controversy was discussed and consensus between the authors was reached with the involvement of a third author (PS). (Fig. 1)

Data on the study characteristics and design, level of evidence (LOE), demographic parameters as well as treatment relevant data were extracted in a spreadsheet. The primary outcomes were mobility, pain, joint function (e.g. neutral zero range of motion) and patient reported outcome measures (PROMs) (e.g. KOOS, Lysholm, IKDC Child) [15, 16]. Other endpoints included the type of avulsion fracture described, performed diagnostics, type of procedure performed (non-surgical or surgical) and need for revision surgery.

## Results

**Fig. 2:**
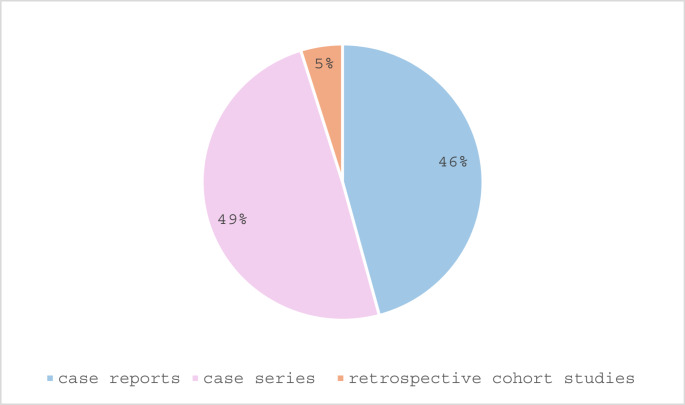
Division of the included manuscripts according to type of study

**Fig. 3:**
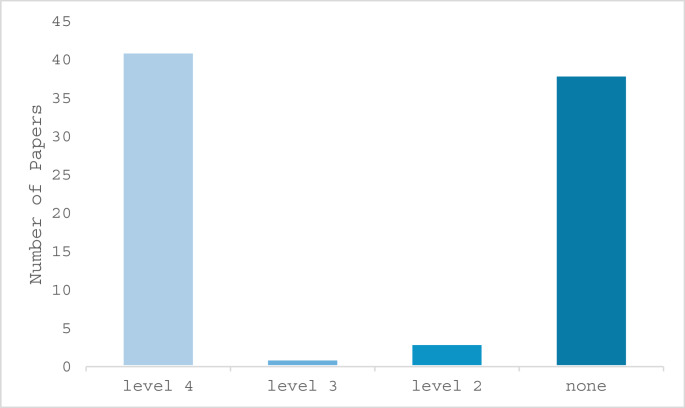
Division of the included manuscripts according to CEBM level of evidence

A total of 83 studies were included for analysis including 38 case reports (45.8%), 41 case series (including prospective and retrospective; 49.4%) and 4 retrospective cohort studies (4.8%). (Fig. 2) The CEBM level of evidence was 4 in 41 (49.4%) of the studies, 3 in one study (1.2%) and 2b in 3 (3.6%) studies. (Fig. 3) In total 12 of the studies (14.5%) were presenting both adult and pediatric cases [17–29], and 13 (15.7%) presented comparative analyses [19, 20, 23, 28–37]. A meta-analysis could not be performed due to the great heterogeneity of the studies and the limited amount high evidence level studies.

The earliest publication was from 1981 [38], and the most recent were from 2023 [36, 39, 40]. The main affiliation of 32 (38.6%) of the publications was in North America, 29 (34.9%) were from Europe, 19 (22.9%) from Asia, 2 (2.4%) from South America and 1 (1.2%) from Africa.

In total 1676 patients were included in the studies (average: 20.2 patients per study; min: 1 patient; max: 661 patients) with a mean age at trauma of 13.1 years (SD:1.9; min: 7; max: 17). 72% of the included patients were male. In 49 studies (59.0%) only male patients were presented. In 75 of the included studies (90.4%) a minimum follow-up time was stated, with a mean of 16.5 months (SD: 13.27 months; min: 0 months; max: 84 months). In 69 of the 83 studies (83.1%) a mean follow-up was stated, with a mean of 24.9 months (SD: 20.48 months; min: 0 months; max: 105.6 months).

Table 1 Shows an overview of the avulsion types presented in the included papers.

**Table 1:** Avulsion types described in the included papers

Avulsion Type	Number of manuscripts, %	Number of patients	References
tibia eminence fractures	27, 32.5%	1278	[17–23, 26–29, 32, 33, 36, 37, 41–52]
unilateral tibia tubercle avulsion fractures	15, 18.1%	336	[30, 31, 34, 35, 53–63]
tibia tubercle fracture with concomitant avulsion of the patella	11, 13.3%	15	[64–74]
tibial avulsion fracture of the PCL	9, 10.8%	15	[11, 24, 25, 75–80]
bilateral tibia tubercle avulsion fractures	5, 6.0%	8	[39, 81–84]
femoral avulsion of the ACL	3, 3.6%	3	[85–87]
avulsion fractures from the femoral attachment of the posterolateral structures (LCL and popliteus)	3, 3.6%	8	[88–90]
tibia tubercle avulsion fracture with ACL rupture and meniscal tear	2, 2.4%	2	[91, 92]
simultaneous avulsion fracture of both the femoral and tibial insertion of ACL	2, 2.4%	2	[38, 93]
avulsion fracture of the MCL	2, 2.4%	2	[94, 95]
femoral avulsion fracture of the PCL	2, 2.4%	3	[40, 96]
chronic femoral avulsion of posteromedial corner with concomitant full ACL rupture	1, 1.2%	1	[97]
sleeve fracture tibial metaphysis	1, 1.2%	3	[98]

In total, 21 of the studies presented patient related outcome measures (PROMS) [17, 19–23, 26–29, 32, 33, 43, 53, 75, 77, 78, 81, 89, 94, 96]. On average these studies used 2 PROMS (SD: 1.00; min: 1; max: 4). The most used PROMS were the Lysholm Knee Score, which was used in 16 studies, the IKDC which was used in eleven studies and the Tegner Activity Score which was used in seven studies. Furthermore 59 studies evaluated full ROM restoration, 50 studies evaluated persistent stiffness of the knee, 48 studies evaluated full sport activity restoration and 42 evaluated persistent pain.

### Tibia eminence fractures

The literature search identified 27 studies that explored tibia eminence fractures [17–23, 26–29, 32, 33, 36, 37, 41–52]. These studies included a total of 1278 patients (min:1; max: 661 patients) with an average age of 11.9 years (SD: 1.6; min: 8 years; max: 16 years) [17–23, 26–29, 32, 33, 36, 37, 41–52]. The majority of the patients were male (65.3%). The mean minimum time of follow-up was 17.0 months (SD: 8.4 months; min 4 months; max: 38.4 months), while the average mean time of follow-up was 35.6 months (SD: 23.1 months; min 4 months; max: 105.6 months). The studies included three case studies [43, 45, 47], 22 case series [17–23, 26–29, 32, 37, 41, 42, 44, 46, 48–52], and two retrospective cohort studies [36, 85]. In total 19 of the studies described surgical treatment of the patients [17, 19–23, 26–29, 32, 36, 41, 43, 45–48, 50]. The surgical methods described were retrograde screw fixation as well as open or arthroscopic suture fixation [17, 19–23, 26–29, 32, 36, 41, 43, 45–48, 50]. Eight of the studies described both surgical and non-surgical treatment of the patients [18, 33, 37, 42, 44, 49, 51, 52]. Three of the studies did not specify the non-surgical approach [37, 49, 52]. The other five studies described immobilization in casts for different time spans [18, 33, 42, 44, 51]. The surgical treatment described in the studies were open or arthroscopic suture fixation [18, 33, 37, 42, 44, 49, 51]. 

Shimberg et al. found that the classification of type 1 fractures of the tibia eminence remains a notable challenge. A significant number of the 48 patients included in the study had accompanying injuries, leading to the need for surgical intervention in over 25% of the cases [37]. Three of the studies further evaluated the incidence of concomitant meniscal and chondral injuries in children and adolescents with tibia eminence fractures [50–52]. The studies found that accompanying soft-tissue injuries are as high as nearly 40%, concluding that an MRI should be performed in every patient with tibia eminence fracture [50–52]. Feucht et al. found in particular, that concomitant meniscal tears have a higher incidence in patients with higher Tanner stages [50]. The study by O’Donnell et al., which included 661 patients, found an overall low incidence of growth disturbance following the surgical treatment of tibial eminence fractures. Furthermore, no association was observed between growth disturbance and demographic factors, fracture characteristics, surgical technique, hardware type, or anatomical placement (i.e., transphyseal vs. physeal-sparing fixation) [36].

### Tibia tubercle fractures

Fifteen publications in total, containing 336 patients (min:1; max: 134 patients), investigated pediatric tibia tubercle fractures [30, 31, 34, 35, 53–63]. The studies included four comparative studies [30, 31, 34, 35], and two retrospective cohort studies [30, 35]. The mean age of the patients was 14.4 years (min: 13 years; max: 16 years), with patients being predominantly male (94.6%). The mean minimum time of follow-up was 16.7 months (SD: 12.3 months; min: 2 months; max: 36 months), while the average mean time of follow-up was 28 months (SD: 29.7 months; min: 5 months; max: 56 months). 10 of the studies, including 280 patients, described surgical treatment of the tibia tubercle fracture using screw fixation [30, 35, 54–56, 58, 60–63]. Five of the studies included both patients treated surgically or non-surgically [31, 34, 53, 57, 59]. Non-surgical treatment involved immobilization in a cast for at least 6 weeks while surgical treatment involved screw fixation [31, 34, 53, 57, 59]. In their retrospective cohort study including 134 patients, Huang et al. showed that in similar patient cohorts, initiating knee motion before four weeks was safe and yielded comparable outcomes to traditional immobilization (4–6 weeks). Given the benefits for patients and caregivers, providers should consider more progressive postoperative rehabilitation protocols for operatively treated [35]. Arkader et al. found no significant differences in healing, downtimes for activities, or complication rates between unicortical and bicortical fixation for displaced tibial tubercle avulsion fractures in young athletes. Given the comparable outcomes and low complication rates, unicortical fixation appears to be a sufficient and less invasive option for surgical management [30].

### Tibia tubercle fracture with concomitant avulsion of the patella

Eleven publications including 15 patients (92.9% male; mean age 14.4 years) described the treatment of combined tibia tubercle fractures with avulsion fractures of the patella (retraction of a small bony fragment from the patella, along with a larger portion of cartilage and periosteum) [64–74]. The studies included three case series [64, 66, 69] and eight case reports [65, 67, 68, 70–74]. One case study described a case with bifocal avulsion of the patella [72]. The average mean time of follow-up was 16.1 months (SD: 15.5 months; min: 4 months; max: 54 months). All studies described the diagnostic use of radiographic images. In one study the use of CT for diagnostic purposes was described [72], and two studies described the use of MRI [69, 70]. All patients received surgical treatment, with none of the studies reported the need for a revision. Four patients received planned removal of the material [64, 66, 73]. All studies described surgical management of the tibia tubercle fracture using open reduction with subsequent fixation using screws. Seven studies additionally also described the surgical treatment of the patellar tendon, mostly with suture anchors [64–72, 74]. None of the studies reported on PROMs. Eight studies, including eleven patients, reported on ROM at follow-up [64, 66–68, 71–74]. Full ROM was achieved by ten patients (90.9%) [64, 66–68, 71, 72, 74]. Six studies, including nine patients reported on pain levels. A pain-free situation was reached by the majority of the patients (88.8%) [64, 66–68, 72, 73]. Nine studies, including 13 patients, reported on return to sports. Unlimited return to sports was reached by 84.6% of the patients [64, 66–69, 71, 72, 74].

### Avulsion fracture of the PCL (tibial and femoral)

Nine publications, including 15 patients (93.3% male; mean age 13.0 years) described the treatment of tibial avulsions of the PCL [11, 24, 25, 75–80]. The studies included two case series [11, 75] and six case reports [24, 76–80] as well as one technical note [25]. The average mean time of follow-up was 24.7 months (SD: 27.7 months; min: 0 months; max: 84 months). All studies described the diagnostic use of radiographic images. Two studies used CT-scans in the diagnostic process [25, 75] and eight studies used MRI scans for all patients [11, 24, 25, 75–78, 80]. All patients received surgical treatment. Three studies, including eight patients, presented surgical therapy with arthroscopic suture fixation [25, 75, 80]. None of the studies reported the need for a revision. Six studies, including twelve patients, reported whether full ROM and freedom of pain were achieved after treatment [11, 75–79]. All patients achieved full ROM and freedom of pain. Seven studies, including 13 patients, reported whether return to sports was reached [11, 75–80]. All patients, except one, were able to return to full sports activity.

Two studies, including three patients (100.0% male, mean age 8.0 years) described the treatment of femoral avulsions of the PCL [40, 96]. The studies included one case report in which the patient showed a concomitant meniscal tear [96] and one case series with two patients [40]. The case presented in the report had a follow-up time of 24 months. The case series had a minimum follow-up time of 13 months and a mean follow-up of 18.5 months. All patients received surgical treatment. Two of the patients received arthroscopic suture fixation and one patient received fixation with a screw. No revisions or complications were reported by the authors. All patients showed full ROM and freedom of pain and restored full sports activity at the final follow-up [40, 96]. A Lysholm score of 84 was reached in the patient described in the case report [96].

### Bilateral tibia tubercle avulsion fractures

Five studies, including eight patients (87.5% male; mean age 14.7 years) reported on bilateral tibia tubercle avulsion fractures (tibia tubercle fractures of both knees concurrently) [39, 81–84]. The studies included one case series [81], three case reports [82–84] and one technical note [39]. The average mean time of follow-up was 8.8 months (SD: 9.9 months; min: 0 months; max: 24 months). All studies used radiographs [39, 81–84], and one study used CT imaging in the diagnostic process [39]. All patients received surgical treatment with open reduction and fixation of the tibia tubercle with either K-wires or cannulated screws. One study reported one patient that developed post-traumatic genu valgum, which was fully corrected by epiphysiodesis in the proximal medial tibia [81]. Yue et al. reported that a fasciotomy had to be performed in their patient due to compartment syndrome [84]. Four studies, including seven patients, reported on full ROM restoration and whether return to sports was possible [81–84]. In the case series there was no change in the Tegner Activity Scale or the Lysholm-Gillquist Score and all patients regained knee function comparable to the pre-injury level [81].

### Combined injuries of ACL avulsion fractures

Two studies were case reports on simultaneous avulsion fractures of both the femoral and tibial insertion of the ACL (100.0% male; mean age 12.0 years), which both had a follow-up of twelve months [38, 93]. One case report described the treatment of this injury in a knee brace for 6 weeks [93]. The other case report described the treatment with suture fixation [38]. In both studies the patient had full ROM and no stiffness or laxity at the end of the follow-up period.

Two further case reports described tibia tubercle avulsion fractures with associated ACL rupture and meniscal tear (100.0% male; mean age 15.5 years) [91, 92]. The average follow-up was 27 months. In both cases the tibia tubercle fracture was reduced openly and the meniscus and ACL fixed with sutures. In the case report by Falster et al. the patient had subjective instability at the 36 months of follow-up [92]. The case report did not mention any other endpoints such as ROM, pain or stiffness. In the case report by Lipscomb et al. the patient described had no stiffness or instability after 18 months. Furthermore the authors reported full ROM restoration and return to sport [91]. 

Three studies, all being case reports, described the treatment of femoral avulsion fractures of the ACL (66.6% male, mean age: 11.7 years) [85–87]. One study described a femoral ACL avulsion with concomitant avulsion of anteromedial and posterolateral bundle [86]. The mean follow-up was 20.3 months. All three patients received arthroscopically assisted reduction and suture fixation of the femoral avulsion. All the patients restored full ROM and sports activity. None of the patients was reported to have pain, stiffness or laxity [85–87].

## Discussion

This systematic review aimed to consolidate the current knowledge surrounding pediatric avulsion fractures of the knee, a rare but significant injury due to its potential long-term impact on growth and joint function. Although tibial eminence fractures and tibial tubercle avulsion fractures have been well studied and documented, other rarely occurring types of avulsion fractures, such as femoral avulsions of the posterior cruciate ligament or avulsion fractures of the collateral ligaments, remain underreported in the literature.

Tibia eminence fractures and tibia tubercle fractures are common injuries in children and adolescents, and they have been described with various treatment approaches in the literature [99]. Several surgical methods were described, including antegrade screw fixation and open or arthroscopic suture fixation. Notably, 19 out of the 27 studies described surgical treatment [17, 19–23, 26–29, 32, 36, 41, 43, 45–48, 50], while others included both surgical and non-surgical approaches [18, 33, 37, 42, 44, 49, 51, 52]. The results, particularly concerning the low incidence of growth disturbances following surgical treatment, confirm the effectiveness of the surgeries [36]. No significant association was found between demographic factors and growth disturbances, underscoring the safety of surgical interventions in this patient group [36]. Nonetheless, careful clinical and radiological follow-up and monitoring remain necessary. A significant aspect highlighted in the studies on tibia eminence fractures is the high incidence of concomitant injuries such as meniscal and cartilage injuries, identified in up to 40% of patients [37, 50–52]. This leads to the recommendation of performing an MRI in all patients with tibia eminence fractures to detect and address any accompanying injuries early. Notably, the study by Feucht et al. found that the incidence of meniscal tears was higher in patients at more advanced Tanner stages, indicating an increased risk of injury during puberty [50]. 

In regard to tibia tubercle fractures, the 15 studies also showed a high prevalence of male patients [30, 31, 34, 35, 53–63]. Surgical treatment was similarly widespread, with screw fixation being the most common method [30, 35, 54–56, 58, 60–63]. Interestingly, the retrospective cohort study by Huang et al. showed that early knee mobilization, less than four weeks post-surgery, yielded outcomes comparable to traditional immobilization (4–6 weeks) [35]. This could be significant for clinical practice, as accelerated rehabilitation could benefit both patients and caregivers.

The analysis of studies on tibia tubercle fractures with concomitant patellar fractures reveals that all patients received surgical treatment, with open reduction and screw fixation of the tibia tubercle fracture [64–74]. Most patients achieved full range of motion and freedom of pain, underscoring the efficacy of surgical therapy for these complex injuries.

The treatment of PCL avulsion fractures, both tibial and femoral, was investigated in nine studies with 15 patients [11, 24, 25, 75–80]. All patients underwent surgical treatment, with results showing complete restoration of range of motion and sports activity [11, 24, 25, 75–80]. Notably, all studies used arthroscopic suture fixation, which is considered the standard procedure and provided excellent outcomes in terms of functional recovery.

Our study has several limitations. First, the overall level of evidence in the literature remains low, with most included studies being case reports and case series, limiting the generalizability of the findings. Second, the heterogeneity of study designs, patient populations, and outcome measures made quantitative analyses difficult. Third, the review is limited to publications in English and German, potentially excluding relevant data from other languages. Lastly, the studies included lacked uniformity in terms of follow-up duration, with some reporting very short follow-up periods, making it challenging to assess the true long-term outcomes of these injuries. Furthermore, the heterogeneity of the included studies, both in terms of fracture types and treatment approaches, limited the ability to conduct a meta-analysis in this review. This reflects the need for more high-quality studies and standardized reporting of outcomes. Moving forward, collaboration across institutions and countries could foster the development of comprehensive registries for pediatric avulsion fractures, enabling more detailed and systematic data collection on these injuries.

## Data Availability

No datasets were generated or analysed during the current study.
